# A novel *de novo *mutation in the serine-threonine kinase *STK11 *gene in a Korean patient with Peutz-Jeghers syndrome

**DOI:** 10.1186/1471-2350-9-44

**Published:** 2008-05-22

**Authors:** Jong-Ha Yoo, Jee-Hyoung Yoo, Yoon-Jung Choi, Jung-Gu Kang, Young-Kyu Sun, Chang-Seok Ki, Kyung-A Lee, Jong-Rak Choi

**Affiliations:** 1 Department of Laboratory Medicine, National Health Insurance Corporation Ilsan Hospital, Goyang, Korea; 2 Department of Pediatrics, National Health Insurance Corporation Ilsan Hospital, Goyang, Korea; 3 Department of Pathology, National Health Insurance Corporation Ilsan Hospital, Goyang, Korea; 4 Department of Surgery, National Health Insurance Corporation Ilsan Hospital, Goyang, Korea; 5 Department of Laboratory Medicine, Samsung Medical center, Sungkyunkwan University School of Medicine, Seoul, Korea; 6 Department of Laboratory Medicine, Yonsei University College of Medicine, Seoul, Korea

## Abstract

**Background:**

Peutz-Jeghers syndrome (PJS) is an unusual autosomal dominant disorder characterized by mucocutaneous pigmentation and multiple gastrointestinal hamartomatous polyps. Patients with PJS are at an increased risk of developing multi-organ cancer, most frequently those involving the gastrointestinal tract. Germline mutation of the *STK11 *gene, which encodes a serine-threonine kinase, is responsible for PJS.

**Methods:**

Using DNA samples obtained from the patient and his family members, we sequenced nine exons and flanking intron regions of the *STK11 *gene using polymerase chain reaction (PCR) and direct sequencing.

**Results:**

Sequencing of the *STK11 *gene in the proband of the family revealed a novel 1-base pair deletion of guanine (G) in exon 6 (c.826delG; Gly276AlafsX11). This mutation resulted in a premature termination at codon 286, predicting a partial loss of the kinase domain and complete loss of the C-terminal domain. We did not observe this mutation in both parents of the PJS patient. Therefore, it is considered a novel *de novo *mutation.

**Conclusion:**

The results presented herein enlarge the spectrum of mutations of the *STK11 *gene by identifying a novel *de novo *mutation in a PJS patient and further support the hypothesis that *STK11 *mutations are disease-causing mutations for PJS with or without a positive family history.

## Background

Peutz-Jeghers syndrome (PJS; OMIM 175200) is a rare, autosomal dominant disorder characterized by melanocytic macules of the lips, buccal mucosa, and digits, along with multiple gastrointestinal hamartomatous polyps, frequently in the small intestine [1,2]. Patients with PJS are at an increased risk of developing gastrointestinal cancer and extraintestinal neoplasms involving organs such as the ovaries, testes, breasts, pancreas, lungs, or uterine cervix [3].

Currently, only mutations in the gene *STK11 *(also known as *LKB1*; OMIM 602216) at chromosome 19p13.3 have been identified as a cause of PJS [4,5]. The human *STK11 *gene encodes a 433 amino acid serine-threonine kinase. *STK11 *is known to be located both in the nucleus and the cytoplasm of all human tissues [6], and orthologs include mouse *LKb1 *[7], *XEEK1 *(*Xenopus *egg and embryo kinase 1) [8], *Caenorhabditis elegans *partitioning defective gene 4 (*par-4*) [9], and *drosophila Lkb1 *[10].

Loss of the normal allele has been observed in polyps from patients with PJS, and loss of heterozygosity (LOH) has been noted to occur in some tumor tissues, suggesting that *STK11 *is a tumor suppressor gene [11]. *STK11 *has been shown to cause apoptosis in intestinal epithelial cells, and is physically associated with p53, regulating specific p53-dependent apoptosis pathways [12]. *STK11 *is also known to have effects on G1 cell cycle arrest [13], TGF-β signaling [14], polarity [15], and phosphorylating and activating the AMP-activated protein kinase (AMPK) [16].

Screening for point mutations and large deletions by direct sequencing or by multiplex ligation-dependent probe amplification (MLPA) increased the mutation detection rate in the *STK11 *gene up to 94% [17]. To date, more than 200 different mutations in the *STK11 *gene have been reported at the Human Gene Mutation Database (HGMD) website  and most are small insertions/deletions or missense/nonsense mutations.

We report on a Korean PJS patient with a novel *STK11 *mutation. During molecular genetic testing for *STK11 *mutation, we detected a novel small deletion in exon 6, causing a premature stop codon. This mutation was absent in both parents of the patient and was thus a *de novo *mutation.

## Methods

### Subjects

**Figure 1:**
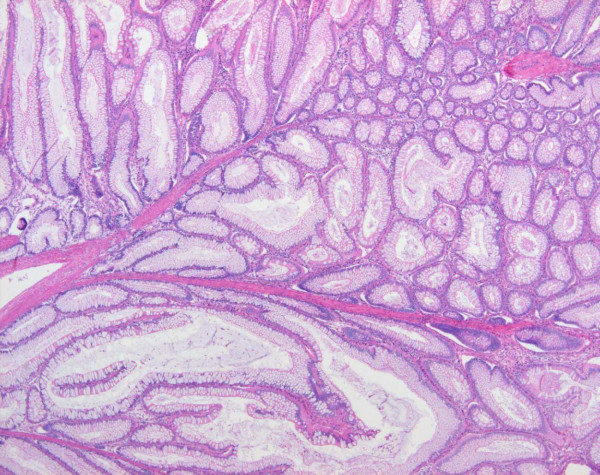
**Pathologic findings of the Peutz-Jeghers polyp**. The colonic polyp shows hyperplastic mucosal epithelium and arborizing pattern of smooth muscle, consistent with a hamartomatous polyp (Hematoxylin-eosin stain, 200×).

**Figure 2:**
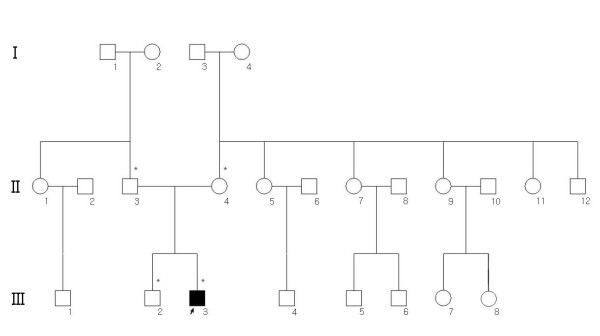
**Pedigree of the family with PJS**. *Circle*, female; *square*, male; *black symbol*, affected. Asterisk (*) indicates the family member who was available for genetic analysis.

The proband was a 13-year-old Korean male. He was diagnosed clinically with PJS at six years of age based on the presence of characteristic mucocutaneous pigmentation of the lips and buccal mucosa, and gastrointestinal hamartomatous polyps after polypectomy (Figure 1). After obtaining informed consent, blood samples were collected from four family members including the patient. Brother and parents of the patient did not consent to endoscopic examinations for evaluation of polyps. Relatives of the patient had no known medical conditions, including mucocutaneous pigmentation or malignancies, and no further study was assessed (Figure 2).

### Mutation studies

Four family members including the patient were included in this study after obtaining informed consent. The genomic DNA was isolated from peripheral blood leukocytes using the Wizard Genomic DNA Purification Kit according to the manufacturer's instructions (Promega, Madison, WI, USA). The polyp tissue was not collected for DNA analysis. The *STK11 *gene was amplified via PCR [by using the appropriate primers as designed by the authors (available upon request)] and a thermal cycler (Model 9700; Applied Biosystems, Foster City, CA, USA). Direct sequencing of all nine coding exons along with the flanking intron regions of the *STK11 *gene was performed with the Big Dye Terminator Cycle Sequencing Ready Reaction Kit (Applied Biosystems) in conjunction with an ABI Prism 3100 automated genetic analyzer (Applied Biosystems).

## Results

**Figure 3:**
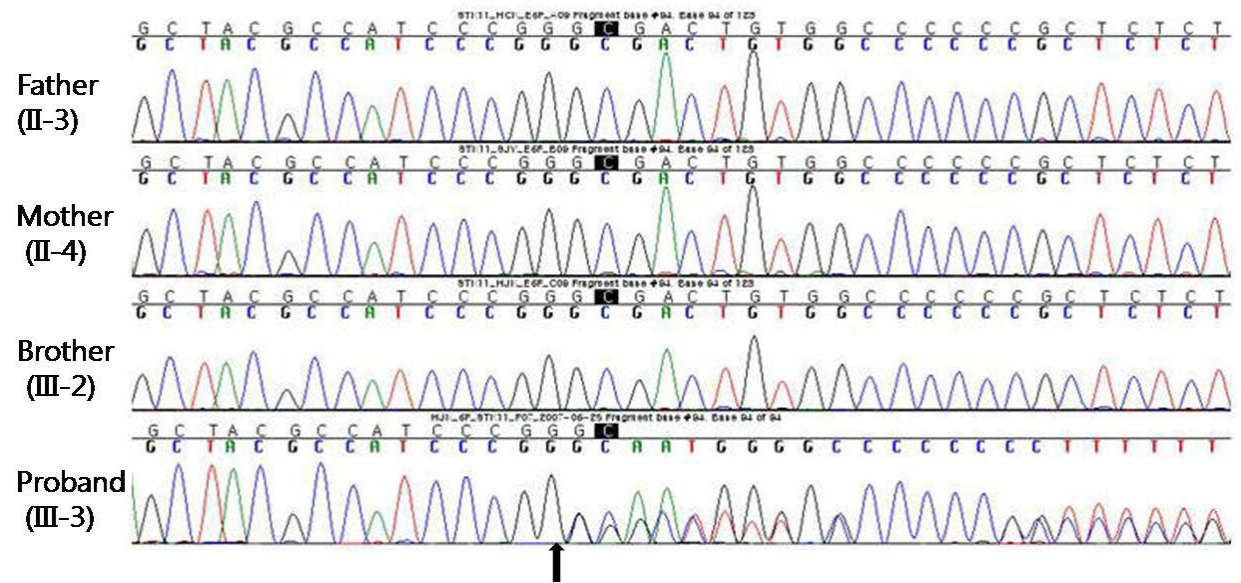
**Identification of the STK11 gene mutation**. Direct sequencing of the proband demonstrated a 1-bp deletion (c.826delG) in exon 6 of the *STK11 *gene, resulting in a frameshift deletion mutation (p.Gly276AlafsX11). The proband's family members did not have the mutation. The localization of the deletion is indicated by the arrow.

Direct sequencing analysis of the proband demonstrated a heterozygous 1-bp deletion of guanine (G) (c.826delG; Gly276AlafsX11) in exon 6 of the *STK11 *gene, which resulted in a frameshift leading to premature termination of the 433 amino acid protein at the 286^th ^codon, disruption of the kinase domain, and complete loss of carboxyterminal non-catalytic region. This mutation was absent in his family members and 100 control chromosomes (Figure 3).

## Discussion

Germline mutations in the *STK11 *gene on chromosome 19p13.3 have been shown to be the cause of PJS [4,5]. Recent study suggests that the *STK11 *mutation detection rate was above 90% [17]. However, some families with PJS have shown linkage to chromosomal region 19q13.4 [18] and 6 [19]. Human *STK11 *consists of nine coding exons with a 433 amino acid coding sequence, and one non-coding exon 10 that spans 23 kb [6,7]. The *STK11 *protein is mainly comprised of three major domains: the N-terminal non-catalytic domain containing the nuclear localization signal, the catalytic kinase domain important for ATP binding, and the carboxyterminal non-catalytic regulatory domain containing prenylation motif (CAAX-box). Codons 49–309 encode the catalytic kinase domain. The C-terminal non-catalytic region of the *STK11 *protein is encoded by exon 8 and 9 and encompasses amino acids 309–433.

The patient recruited in this study fulfilled the well-established clinical diagnostic criteria for PJS [20]. The criteria include histopathologically proven hamartomas together with classical mucocutaneous hyperpigmentation and small-bowel polyposis. Therefore, the possibility that this patient is affected with hamartomatous polyposis syndromes other than PJS is highly unlikely. Such syndromes include juvenile polyposis syndrome, *PTEN *hamartoma tumor syndrome, and Carney complex. In our study, we sequenced the *STK11 *gene in this patient with PJS. We identified a novel heterozygous 1-bp deletion (c.826delG; Gly276AlafsX11) in exon 6 of the *STK11 *gene, which resulted in a frameshift leading to premature termination of the 433 amino acid protein at the 286^th ^codon. This mutation was not detected in the *STK11 *sequencing analysis of his family members, indicating that Gly276AlafsX11 is a novel *de novo *mutation.

The Gly276AlafsX11 mutation is located in the catalytic kinase domain of *STK11 *protein, so we hypothesize that this mutation may lead to partial loss of the kinase domain and complete loss of the C-terminal domain. This mutation is novel, but a similar effect of the mutation in the *STK11 *protein was reported recently [21]. Loss of *STK11 *protein kinase activity associated with loss of growth suppression function was reported in some mutations in *STK11 *associated with PJS [4,22]. Thus, development of the PJS phenotypes is believed to be due to the elimination of the kinase activity of *STK11 *[23]. The C-terminal domain of *STK11 *is important for the control of both the AMPK pathway and cell polarity [15]. Mutations leading to loss of the C-terminal domain of *STK11*, as observed in this case, lead to loss of cell polarity, resulting in the development of malignancies. Taken together, these data suggest that the *STK11 *mutation in exon 6 may contribute to polyp formation and tumorgenesis through various mechanisms such as loss of growth arrest, apoptosis, and loss of cell polarity. Further studies will be needed to address these questions.

Although an increased cancer risk in patients with PJS is well established [3,24], data on genotype-phenotype correlation is lacking. Schumacher *et al*. studied 146 PJS patients and determined that inframe deletions, splice site mutations, and missense mutations in the catalytic kinase domain were rarely associated with cancer. However, it was felt that missense mutations in the C-terminal domain were more frequently associated with malignancies [22]. Recently, however, Hearle *et al*. studied 419 PJS patients and determined that the type or site of the *STK11 *mutation did not significantly influence cancer risk [25]. Restricted by the few published papers on this topic, the genotype-phenotype correlation remains to be further investigated.

## Conclusion

We enlarged the spectrum of mutations of the *STK11 *gene by identifying a novel mutation in a Korean patient with PJS. Because of the increased risk of PJS in patients with multi-organ cancers, molecular diagnosis will be an important factor in genetic counseling, clinical management of patients, and tumor screening.

## List of abbreviations

PJS: Peutz-Jeghers syndrome; STK11/LKB1: serine-threonine kinase 11; XEEK1: *Xenopus *egg and embryo kinase 1; LOH: loss of heterozygosity; AMPK: AMP activated protein kinase; MLPA: multiplex ligation-dependent probe amplification.

## Competing interests

The authors declare that they have no competing interests.

## Authors' contributions

JY recruited all the subjects investigated, carried out the molecular genetic studies, and drafted the manuscript. Y–KS, K–AL, and C–SK helped with the experiments. JY, Y–JC, and J–GK diagnosed the patient and participated in the editing of the manuscript. J–RC designed and supervised the study. All authors read and approved the final manuscript.

## Pre-publication history

The pre-publication history for this paper can be accessed here:


